# Antibacterial effects of *Lactobacillus* isolates of curd and human milk origin against food-borne and human pathogens

**DOI:** 10.1007/s13205-016-0591-7

**Published:** 2017-04-11

**Authors:** Chetan Sharma, Brij Pal Singh, Nishchal Thakur, Sachin Gulati, Sanjolly Gupta, Santosh Kumar Mishra, Harsh Panwar

**Affiliations:** 1 Department of Dairy Microbiology, College of Dairy Science and Technology, Guru Angad Dev Veterinary and Animal Sciences University (GADVASU), Ludhiana, Punjab 141004 India; 2 School of Public Health and Zoonoses, Guru Angad Dev Veterinary and Animal Sciences University (GADVASU), Ludhiana, Punjab 141004 India

**Keywords:** *Lactobacillus*, Antibacterial activity, Pathogens, Probiotics, Cell-free supernatant

## Abstract

This study was undertaken to assess the antibacterial efficacy of lactobacilli isolated from curd and human milk samples. Identities of thirty-one different lactobacilli (20 from curd and 11 from human milk) were confirmed by genus-specific PCR and 16S rRNA-based sequencing. These strains belonged to five species, *Lactobacillus casei*, *L. delbrueckii*, *L. fermentum*, *L. plantarum,* and *L. pentosus*. Antibacterial activities of cell-free supernatants (CFSs) of all the *Lactobacillus* isolates were estimated through standard agar-well diffusion assay, against commonly occurring food-borne and clinically important human pathogens. None of the lactobacilli cell-free supernatant (CFS) exhibited inhibitory activity against four pathogens, namely *Staphylococcus aureus*, *Listeria monocytogenes, Escherichia coli,* and *Klebsiella pneumoniae. Bacillus cereus*, *Salmonella enterica* serovar Typhi, and *Shigella flexneri* were moderately inhibited by majority of CFSs, whereas, weak activity was observed against *Pseudomonas aeruginosa* and *Proteus mirabilis*. CFS of some of the curd isolates displayed antagonistic activity against *Streptococcus mutans*; however, human milk lactobacilli did not displayed any inhibitory activity against them. As expected, Nisin (Nisaplin^®^) showed inhibitory activity against Gram-positive, *S. aureus*, *B. cereus,* and *L. monocytogenes.* Interestingly, few of the examined CFSs exhibited inhibitory activities against both Gram-positive and Gram-negative pathogens. Findings from this study support the possibility to explore the tested lactobacilli and their CFSs as natural bio-preservatives, alone or in combination with approved bacteriocins in food and pharma formulations after validating their safety.

## Introduction

The perception of functional food with physiologically active components has been recognized in the history of mankind. Still, the renewed interest of consumers toward ready to eat, minimally processed and preserved food with additional physiological benefit has raised challenges for the food industry. Thereby, within the last few decades, the use of microorganism and their products for the preservation of food has largely arrested the interest of manufacturers and prompted food technologists/researchers to explore the application of natural compounds for the preservation of food products (Georgieva et al. 2015). To compensate this need, lactic acid bacteria (LAB) intends to be an appropriate candidate due to its varied antimicrobial, bio-therapeutic and preservation properties (Arena et al. 2016; Mangiapane et al. 2015). LABs are widespread in nature and reside in a variety of natural habitats, ranging from plants to the mammalian oral, gastrointestinal and vaginal cavities (Benavides et al. 2016). There is increasing interest in certain LABs, especially *Lactobacillus* and *Bifidobacterium* spp. which are extensively explored in the area of food, either as technological starters in the fermented products, as probiotics, or as potential food preservatives (Altay et al. 2013). Additionally, they help in maintaining the normal intestinal permeability and restoration of gut microflora, enhancement of the intestine’s immunological barrier functions and improvement of the intestinal inflammatory response (Jose et al. 2015). Many studies and reports have also documented the clinical importance of beneficial microbes in different clinical ailments, such as allergic pathologies (atopic eczema and rhinitis), diarrhea, necrotizing enterocolitis, inflammatory bowel disease, type 2 diabetes, and viral infections (Panwar et al. 2013; Presti et al. 2015; Thakur et al. 2016).

The antimicrobial effect of lactobacilli is primarily linked to the production of organic acids, such as lactic acid, acetic acid, propionic acid, and sometimes hydrogen peroxide, bacteriocins, and antimicrobial peptides (AMPs) with a variable range of action (Cortes-Zavaleta et al. 2014; Gemechu 2015). Strains of lactobacilli can produce organic acids through heterofermentative pathways. These acids may perhaps interact with the cell membrane and induce intracellular acidification and protein denaturation. The antibacterial effect of lactic acid is probably due to the physiological and morphological changes induced in the bacterial cytoplasmic membrane, resulting in leakage of cytoplasmic contents (Wang et al. 2015). Hydrogen peroxide can act as precursor to the production of bactericidal free radicals (superoxide and hydroxyl), leading to DNA damage and peroxidation of membrane lipids enhancing their membrane permeability (Aminnezhad et al. 2015). Nisin, the most frequently referenced bacteriocin is known to inhibit cell wall synthesis and induce pore formation, which rapidly kills cells. LAB-derived bacteriocins are also known to target bacterial membrane integrity and septum formation during mitosis (Cavera et al. 2015).

Beneficial effects of lactobacilli, including inhibition of Gram-positive and Gram-negative pathogenic and spoilage bacteria have been reported by many researchers (Song et al. 2014; Zhu et al. 2014). The therapeutic role of lactobacilli in controlling the infections caused by *Pseudomonas aeruginosa*, *Staphylococcus aureus* and *Salmonella* spp. has been reported. Combined action of *Lactobacillus* and antibiotic(s) has been shown to be successful in the management of *Helicobacter pylori* infection (Homan and Orel 2015; Safavi et al. 2016). A combination therapy, including probiotic and antibiotic may offer better antimicrobial activity and lessen the dose of antibiotic required. Further, it may also help in replenishment of the intestinal flora thereby providing benefit to the host and abating the side effects of antibiotics (Aminnezhad et al. 2015).

Outbreaks related to food-borne diseases associated with the consumption of fresh and minimally processed fruits and vegetables, primarily due to *E. coli*, *Salmonella* spp., *S. aureus,* and *L. monocytogenes*, have increased dramatically. Presently, modified atmosphere packaging (MAP), chemical preservatives, and refrigeration are among the most preferred tools for extending the shelf-life of food items (Siroli et al. 2015; Li et al. 2016). As a result of recent development in probiotic research, in terms of their efficacy, mechanism of action, and their role in gut microbiota-host interactions, probiotics offers an innovative approach for development of novel probiotic formulations for the management of specific diseases (Grover et al. 2012). The antagonistic activity of these microorganisms and/or their extracellular antibacterial agents (cell-free supernatants) also offers valuable prospects for their application in food preservation (Kecerova et al. 2004), as feed supplements, or in veterinary medicine (Cortes-Zavaleta et al. 2014). As LAB enjoy the ‘generally recognized as safe’ (GRAS) status, their metabolites have captivated substantial interest as natural drugs in recent years (Reis et al. 2012). Milk, besides harboring several natural antimicrobial components, is a favorable source of several beneficial (commensal, mutualistic and/or potentially probiotic) microorganisms, which also assists in the development and initial colonization of the infant gut (Fernandez et al. 2012; Panwar 2014; Reis et al. 2016). Most of the commercial probiotics to date have been of food/dairy or human fecal origin (Ng et al. 2015). Although there are research findings supporting the antimicrobial efficacy of lactobacilli from dairy products, scanty data are available regarding the antibacterial potential of human milk lactobacilli. In lieu of the above facts, the present study was designed to evaluate the efficacy of CFS of curd and human milk lactobacilli against the commonly occurring food-borne and human pathogens.

## Materials and methods

### Chemicals and re-agents

de-Man Rogosa and Sharpe (MRS), Brain–Heart Infusion (BHI), and Mueller–Hinton agar (MHA) base were procured from Hi-Media labs, Mumbai, India. Nisin (Nisaplin^®^, 10^6^ IU/g) taken as control has been generously gifted by Dr. R.K. Malik, ICAR-NDRI, Karnal.

### Bacterial strains

*Lactobacillus* isolates of curd and human milk origin were taken as subjects for this study. Curd samples were collected from rural households of Punjab, India. Human milk samples were collected from healthy volunteer mothers with no recent history of medication. All the samples were collected in sterile containers and stored on ice until delivery to the laboratory. Lactobacilli were isolated from curd and human milk following standard serial dilution method and plating over selective MRS agar. Identity of Gram-positive, catalase negative rods, i.e., tentative lactobacilli, were further ascertained at molecular level following genus-specific PCR (Panwar et al. 2014) and characterized to species level using 16S rRNA sequencing (Panwar et al. 2016). Lab coding and identity of *Lactobacillus* isolates has been documented in Table 1. Six reference *Lactobacillus* representative cultures, viz., *Lactobacillus fermentum*, *L. helveticus*, *L. plantarum*, *L. bulgaricus*, *L. delbrueckii* subsp. *lactis*, and *L. rhamnosus* were procured from the repository of National Collection of Dairy Cultures (NCDC), ICAR-NDRI, Karnal, India. Two probiotic strains viz. *L. rhamnosus* GG and *L. casei* were procured from American Type Culture Collection (ATCC), USA. Ten pathogenic strains viz. *B. cereus*, *L. monocytogenes*, *S. aureus*, *S. mutans* [Gram-positive]; *S. enterica* serovar Typhi, *E. coli*, *S. flexneri*, *P. aeruginosa*, *P. mirabilis*, and *K. pneumoniae* [Gram-negative] were procured from Microbial Type Culture Collection (MTCC), Chandigarh, India. All the pathogenic strains were handled in class II, type A2 biological safety cabinet. *Lactobacillus* strains were maintained and propagated in MRS broth. Pathogens were maintained and propagated in BHI broth. All the bacterial cultures were preserved as glycerol stocks at −80 °C. Prior to the antibacterial assays, the cultures were sub-cultured thrice in their respective growth medium.

**Table 1:** List of lactobacilli and pathogenic strains used in this study

Code	Identification name	NCBI accession numbers
Curd isolates		
D2	*Lactobacillus plantarum*	KX129818
D4	*L. plantarum*	KX146485
D5	*L. plantarum*	KX943015
D6	*L. delbrueckii* subsp*. indicus*	KX228834
D7	*L. plantarum*	KX228835
D8	*L. plantarum* subsp*. plantarum*	KX228836
D9	*L. delbrueckii* subsp*. indicus*	KX228837
D10	*L. plantarum*	KX228838
D11	*L. fermentum*	KX228839
D12	*L. plantarum*	KX228840
D14	*L. plantarum*	KX943016
D17	*L. plantarum*	KX943017
D18	*L. delbrueckii* subsp*. indicus*	KX228841
D19	*L. delbrueckii* subsp*. delbrueckii*	KX228842
D24	*L. plantarum*	KX943018
D25	*L. plantarum*	KX943019
D26	*L. plantarum*	KX228843
D27	*L. plantarum*	KX943020
D28	*L. fermentum*	KX228844
D29	*L. plantarum*	KX228845
Human milk isolates		
HM1	*L. casei*	KX714820
HM2	*L. plantarum*	KX943021
HM3	*Lactobacillus* sp.	KX301286
HM6	*L. pentosus*	KX301287
HM7	*L. plantarum*	KX301288
HM8	*L. plantarum*	KX301289
HM9	*L. plantarum*	KX714821
HM10	*L. plantarum*	KX301290
HM11	*L. plantarum*	KX714822
HM12	*Lactobacillus* sp.	KX301291
HM13	*L. pentosus*	KX301292
Reference *Lactobacillus* strains		
NCDC 214	*Lactobacillus fermentum*	–
NCDC 194	*L. helveticus*	–
NCDC 20	*L. plantarum*	–
NCDC 27	*L. bulgaricus*	–
NCDC 3	*L. delbrueckii* subsp. *lactis*	–
NCDC 19	*L. rhamnosus*	–
Probiotic *Lactobacillus* strains		
ATCC 53103	*L. rhamnosus* GG	–
ATCC 393	*L. casei*	–
Pathogenic strains		
MTCC 1272	*Bacillus cereus*	–
MTCC 1143	*Listeria monocytogenes*	–
MTCC 96	*Staphylococcus aureus*	–
MTCC 890	*Streptococcus mutans*	–
MTCC 733	*Salmonella enterica* serovar Typhi	–
MTCC 723	*Escherichia coli*	–
MTCC 1457	*Shigella flexneri*	–
MTCC 741	*Pseudomonas aeruginosa*	–
MTCC 425	*Proteus mirabilis*	–
MTCC 109	*Klebsiella pneumoniae*	–

### Assessment of the antibacterial activity

Antibacterial efficacy of CFSs of *Lactobacillus* strains against Gram-positive and Gram-negative pathogens were determined through modified agar-well diffusion assay (Presti et al. 2015; Aneja et al. 2011). CFS of all the tested strains was prepared by harvesting (12,000 g/10 min/4 °C) actively growing, overnight sub-cultured lactobacilli, followed by aseptic collection of the supernatant. The recovered CFS was filter-sterilized by passing through a sterile Uniflo 0.2 μm pore size PVDF Whatman filter. Fresh overnight culture of each pathogen was streaked over BHI agar plate and incubated at 37 °C for 16 h. A minimum of four pure isolated colonies were transferred to sterile normal saline (0.85%) under aseptic conditions. Density of each microbial suspension was adjusted equal to that of 10^6^cfu/ml (0.5McFarland standard) and used as the inoculum for performing agar-well diffusion assay (Andrews 2001). An aliquot of 100 μl of the inoculum of each test pathogen was spread plated over pre-solidified MHA plates. The inoculated agar plates were allowed to dry, and equidistant 8 mm wells were made using a sterile borer. The base of each well was sealed with molten agar medium. 100 μl of CFS was dispensed into each pre-labeled well and un-inoculated MRS broth (100 μl) served as negative control. In order to accelerate the diffusion of bacterial CFS into agar, MHA plates were pre-incubated at 4 °C/1 h, followed by overnight incubation at 37 °C. Nisin (Nisaplin^®^, Danisco), a *Lactococcus lactis* subsp. *lactis* produced natural food additive bacteriocin, was used in this study for comparative analysis. Nisaplin^®^ at a concentration of 10,000 IU/L (Tramer and Fowler 1964) was filter sterilized and assessed for its antibacterial activity, as discussed previously. The antibacterial activity, indicated by the zone of inhibition (ZOI) surrounding the well containing the CFS, was recorded using zone scale (Hi-media). All the tests were performed in triplicate, and the mean values of the diameter of inhibition zones were recorded. The inhibition continuums were recorded as follows: −, no activity (ZOI less than 11); +, weak inhibition (ZOI of 12–15 mm in diameter); ++, moderate inhibition (ZOI of 16–19 mm in diameter); +++, strong inhibition (ZOI of 20–25 mm in diameter) and ++++, very strong inhibition (ZOI more than 25).

## Results and discussion

Antimicrobial activity is a very significant criterion for selection of starter and probiotic culture as they form natural antagonists of potentially harmful bacteria. Therefore, CFSs of *Lactobacillus* strains isolated from curd and human milk were screened for their antagonistic activity against Gram-positive and Gram-negative food-borne and human pathogens. The data for the same have been represented in Tables 2 and 3. CFSs of *Lactobacillus* strains displayed a varied level of inhibitory activity against tested pathogens. Interestingly, *S. aureus, L. monocytogenes, E. coli*, and *K. pneumoniae* were resistant toward CFS of all the *Lactobacillus* strains, i.e., no zone of inhibition was recorded with any of the CFS in their case. Several earlier reports have documented the varied antibacterial activity of CFSs of *Lactobacillus* strains. Similar to our findings, Hawaz (2014) had also observed that filtered supernatants of some of the tested *Lactobacillus* strains did not exhibit any inhibitory activity against *Staphylococcus*, *E. coli*, and *Klebsiella*. Recently, Jose et al. (2015) reported that none of the lactobacilli supernatant could inhibit the growth of *E. coli*. Additionally, few of the dairy isolates failed to display antagonistic activity against *Listeria* species. In contrary, perusal of data in Table 2 revealed that CFSs of lactobacilli exhibited moderate-to-good antagonistic activity against *B. cereus*. Similar to our results, high antagonistic activity against *B. cereus* was reported by other researchers (Bahri et al. 2014; Mahasneh et al. 2015) as well. CFSs of lactobacilli displayed varied antibacterial activities, and this could be attributed to the secretion of different antimicrobial substances or metabolites such as organic acids (lactic and acetic acids), hydrogen peroxide, ethanol, diacetyl, bacteriocins, peptides etc. (Kumar and Kumar 2015).

**Table 2:** Antibacterial profile of CFSs of lactobacilli isolated from curd and human milk samples

Isolates	*S. aureus*	*B. cereus*	*S. mutans*	*L. monocytogenes*	*S. enterica* serovar Typhi	*P. mirabilis*	*S. flexneri*	*P. aeruginosa*	*E. coli*	*K. pneumoniae*
Curd isolates										
D2	−	++	−	−	+	+	++	−	−	−
D4	−	++	−	−	+	+	++	−	−	−
D5	−	++	−	−	+	++	++	−	−	−
D6	−	++	−	−	+	+	++	−	−	−
D7	−	++	−	−	++	+	++	−	−	−
D8	−	++	−	−	+	+	+	+	−	−
D9	−	++	−	−	+	+	+	+	−	−
D10	−	++	−	−	+	+	+	+	−	−
D11	−	++	−	−	+	+	+	+	−	−
D12	−	++	−	−	+	+	+	+	−	−
D14	−	+	+	−	−	−	−	−	−	−
D17	−	+	−	−	+	−	+	−	−	−
D18	−	+	−	−	+	−	+	−	−	−
D19	−	+	+	−	+	−	+	−	−	−
D24	−	+	+	−	+	−	+	+	−	−
D25	−	+	+	−	+	+	+	+	−	−
D26	−	+	+	−	−	−	+	+	−	−
D27	−	+	−	−	+	−	+	−	−	−
D28	−	++	−	−	+	+	+	−	−	−
D29	−	+	−	−	−	−	−	−	−	−
Human milk isolates										
HM1	−	++	−	−	+	−	++	++	−	−
HM2	−	+++	−	−	++	−	++	+	−	−
HM3	−	++	−	−	+	−	+	+	−	−
HM6	−	++	−	−	+	−	++	+	−	−
HM7	−	++	−	−	+	−	++	+	−	−
HM8	−	+	−	−	+	−	−	+	−	−
HM9	−	+	−	−	+	−	−	+	−	−
HM10	−	+	−	−	+	−	−	+	−	−
HM11	−	+	−	−	+	−	−	+	−	−
HM12	−	+	−	−	+	−	+	+	−	−
HM13	−	++	−	−	+	−	++	+	−	−

<11 mm, no activity (−); 12–15 mm, weak inhibition (+); 16–19 mm, moderate/average inhibition (++); 20–24, strong inhibition (+++); >25 mm, very strong inhibition (++++)

**Table 3:** Inhibition spectrum of reference and probiotic lactobacilli CFSs and nisin against the tested pathogens

Reference and probiotic *Lactobacillus* strains	*S. aureus*	*B. cereus*	*S. mutans*	*L. monocytogenes*	*S. enterica* serovar Typhi	*P. mirabilis*	*S. flexneri*	*P. aeruginosa*	*E. coli*	*K. pneumoniae*
*L. delbrueckii* subsp. *lactis*	−	++	−	−	+	−	+	−	−	−
*L. rhamnosus*	−	++	−	−	+	+	++	−	−	−
*L. plantarum*	−	+++	++	−	+	+	+	−	−	−
*L. brevis*	−	++	++	−	−	−	−	−	−	−
*L. helveticus*	−	+	−	−	+	−	+	−	−	−
*L. fermentum*	−	+	−	−	−	−	+	−	−	−
*L. rhamnosus* GG	+	+	−	−	+	−	++	+	−	−
*L. casei*	++	+	−	−	++	−	++	+	−	−
Nisin (Nisaplin^®^)	++	++	−	+	−	−	−	−	−	−

Amongst the curd isolates, weak-to-moderate inhibition was observed against *Bacillus cereus*. Highest inhibition (ZOI ~18 mm) against *B. cereus* was recorded with D2, D4, and D8 closely followed by D7 and D9 (ZOI ~17 mm). Only five of the curd isolates, viz., D14, D19, D24, D25, and D26 exhibited weak activity against *S. mutans*. Among Gram-negative pathogens, *S. enterica* serovar Typhi and *S. flexneri* showed weak-to-moderate sensitivity toward majority of CFSs. *P. mirabilis* and *P. aeruginosa* were also inhibited by few of the lactobacilli supernatants. However, the inhibitory effect was milder. This sensitivity of Gram-negative pathogens can be linked to their thin peptidoglycan cell walls and their susceptibility toward acidic metabolites. Two of the examined strains, D14 and D29, showed mild activity against Gram-positive pathogens. In contrast, they were inactive against all the screened Gram-negative pathogens. Our findings are in agreement with those obtained by Balamurugan et al. (2014), who also reported high antagonistic activity of curd *Lactobacillus* isolates of Indian origin against *Salmonella* Typhimurium and *E. coli*. In another study, Hawaz (2014) also reported moderate activity of curd lactobacilli against *Bacillus* sp. and *Pseudomonas* sp. Few of our curd lactobacilli CFSs displayed antagonistic activity against *S. mutans*, which is in agreement to recent studies that have claimed the antagonistic potential of lactobacilli against *Streptococcus* spp. (Chen et al. 2013; Taheur et al. 2016).

In addition, human milk lactobacilli CFSs also showed varying antagonistic patterns against the tested pathogens. Similar to curd isolates, human milk *Lactobacillus* CFSs were inactive against *S. aureus*, *L. monocytogenes*, *E. coli* and *K. pneumoniae*. Additionally, no inhibitory activity was recorded against *S. mutans* and *P. mirabilis*. Several strains of *E. coli*, *L. monocytogenes* and *S. aureus* have been reported to display inducible cellular resistance against low pH, weak acids and hydrogen peroxide (Brul and Coote 1999; Cotter and Hill 2003); which may be an active factor responsible for resistance exhibited toward the CFSs. In general, *S. aureus* is quite acid resistant and possess multitude of defense mechanisms (Bore et al. 2007), that may be one of the factors mediating resistance toward the CFSs. HM2 (*L. plantarum*) exhibited strong inhibitory activity against *B. cereus,* as depicted with the high zone of inhibition among all the sensitive strains. Both *B. cereus* and *S. enterica* serovar Typhi were sensitive to the CFS of all the human milk isolates. Earlier Olivares et al. (2006) and Kozak et al. (2015) have also documented the antagonistic potential of human milk lactobacilli CFSs against *Salmonella* spp. Among the Gram-negative pathogens, all the isolates displayed weak activity against *P. aeruginosa*. Additionally, *S. flexneri* was inhibited by seven, out of eleven isolates; however, the inhibitory potential was moderate. Our findings are in accordance with Diba et al. (2013), who also assessed the inhibitory potential of human milk lactobacilli against different pathogens. Both *S. aureus* and *E. coli* were reported to be resistant toward lactobacilli CFSs. However, sensitivity was displayed by *Salmonella, Shigella*, and *Pseudomonas* sp. Our results are also in agreement to the findings of Serrano-Nino et al. (2016), where heat-inactivated cell-free broth of tested human milk lactobacilli did not exhibited any inhibitory activity against *L. monocytogenes and S. aureus*.

Among the six reference *Lactobacillus* strains, weak-to-strong inhibitory activity was recorded against *B. cereus*. *L. plantarum* showed highest activity, with a ZOI ~20 mm. This activity can be correlated with HM-2, a human milk *L. plantarum* isolate showing equivalent inhibition. The pathogen inhibitory activity seems to be strain specific, since variable inhibition was recorded even within same species. Among other Gram-positive pathogens, *L. monocytogenes* showed resistance toward CFS of all the reference strains. *S. mutans* was inhibited moderately by both *L. plantarum* and *L. brevis*. Interestingly, *S. aureus* was sensitive toward CFS of *L. rhamnosus* GG and *L. casei*. *L. brevis* failed to inhibit any of the tested Gram-negative pathogens. Gram-negative pathogens, *E. coli* and *K. pneumoniae* showed resistance toward all the reference LAB strains and probiotics. Both, *P. mirabilis* and *P. aeruginosa,* were sensitive to only two, out of six reference lactobacilli CFSs. However, the activity was weak. Further, both *L*. *rhamnosus* GG and *L*. *casei* were inhibitory toward *S.* Typhi, *S. flexneri,* and *P. aeruginosa.* Commercially available bacteriocin, i.e., Nisin showed antagonistic activity against Gram-positive pathogens, except against *S. mutans*. Both *S. aureus* and *B. cereus* were inhibited to a moderate level. No inhibitory activity was observed against Gram-negative pathogens used in the study (Table 3). The antibacterial activity of nisin against several clinically relevant Gram-positive pathogens is well documented. Nisin has found application either as a direct food additive or through food packaging films or coating. The active *Lactobacillus* CFSs may find application as bio-preservative at some point in future. Co-administration of nisin and CFSs may be explored for their synergistic additive effects, after studying their impact over sensory and nutritional attributes of food, without compromising with safety. On similar lines, Aminnezhad et al. (2015) recently evaluated the synergistic action of aminoglycoside antibiotics and CFS from *L. casei* and *L. rhamnosus* against *P. aeruginosa*. A significant inhibitory effect of antibiotic and CFS combination over the growth of *P. aeruginosa* was reported.

Dairy products along with meat and eggs are the most common causes of *Salmonella* spp. mediated food-borne infections (Arques et al. 2015). Interestingly, CFSs of most of our isolates displayed antagonistic activity against *Salmonella enterica* serovar Typhi. Abdel-Daim et al. (2013) and Bahri et al. (2014) also reported strong-to-weak inhibitory activity of *Lactobacillus* supernatants against *Salmonella* spp. Besides, we have also observed the antagonistic activity of *Lactobacillus* CFSs against *P. aeruginosa*, one of the alarming opportunistic pathogen in hospitalized, immuno-compromised, and cystic fibrosis patients. *P. aeruginosa*-mediated infections are often life-threatening and complicated to treat, due to limited susceptibility to commonly practiced antimicrobial drugs (Jamalifar et al. 2011). Our results are in accordance with the findings of Aminnezhad et al. (2015), who also displayed antagonistic activity of lactobacilli CFS against *P. aeruginosa*.

Few of the CFSs of curd and human milk *Lactobacillus* isolates exhibited pathogen inhibitory activity comparable to the probiotic strains. Overall, CFSs of all the tested lactobacilli exhibited strong-to-moderate antagonistic activity against *B. cereus*, which causes severe food poisoning and is frequently isolated from uncooked and unprocessed products such as rice, condiments, vegetables, meat, and milk products. This highly toxic strain is reported to be responsible for food-related fatalities (Arnesen et al. 2008). The application of CFSs of selected lactobacilli as antimicrobial agents could be a promising approach in food preservation. Several antimicrobial peptides produced by LABs have been characterized as potential applicant in food preservation and safety. It is worthwhile revealing that in vitro studies have recommended the prospective and intriguing biomedical applications of CFSs from *Lactobacillus* strains, such as inhibition of cancer metastasis, positive modulation of the intestinal immune response, and cholesterol-lowering properties (Arena et al. 2016). Further, the antimicrobial potency of the proposed combination of lactobacilli CFS and bacteriocin can be useful for designing and developing alternative therapeutic strategies against food-borne and human pathogens. This study highlighted the antibacterial potency of several LAB strains of curd and human milk origin. However, whether the antimicrobial activity exhibited by the CFSs of selected strains was due to the organic acids, fatty acids, active bacteriocin, or any other related substances remains yet to be investigated. Our findings support the hypothesis that these LAB isolates may have application as natural antimicrobial agents in gut and food matrix, and the metabolites produced by these strains could be explored as alternative pharmaceutical compounds with promising therapeutic indices, after identification of their active component, testing their cytotoxic effects and validating safety under in vitro and in vivo models.
